# The Cholinergic and ACE-2-Dependent Anti-Inflammatory Systems in the Lung: New Scenarios Emerging From COVID-19

**DOI:** 10.3389/fphys.2021.653985

**Published:** 2021-05-13

**Authors:** Martina Di Maro, Mauro Cataldi, Mariarosaria Santillo, Martina Chiurazzi, Simona Damiano, Barbara De Conno, Antonio Colantuoni, Bruna Guida

**Affiliations:** ^1^Department of Clinical Medicine and Surgery, Physiology Nutrition Unit, University of Naples Federico II, Naples, Italy; ^2^Department of Neuroscience, Reproductive Sciences and Dentistry, Division of Pharmacology, University of Naples Federico II, Naples, Italy

**Keywords:** severe acute respiratory syndrome coronavirus 2, lung, inflammation, nicotinic acetylcholine receptors, angiotensin-converting enzyme 2

## Abstract

The renin angiotensin system and the cholinergic anti-inflammatory pathway have been recently shown to modulate lung inflammation in patients with COVID-19. We will show how studies performed on this disease are starting to provide evidence that these two anti-inflammatory systems may functionally interact with each other, a mechanism that could have a more general physiological relevance than only COVID-19 infection.

## Introduction

Two anti-inflammatory systems have primary role in controlling inflammation in the lung. The first is based on cholinergic neurotransmission and neuronal-type nicotinic acetylcholine receptors (nAChR) which are nowadays emerging as important players, not only in lung inflammation but also in lung carcinogenesis. The other system is centered on small peptides generated by the Renin-Angiotensin System (RAS) whose role in the control of arterial blood pressure is well known, but recently emerged as important mediators of inflammation not only in the lung but also in other organs, such as the kidney or the cardiovascular system. Recent investigations on the pathogenetic mechanisms of the ongoing devastating COVID-19 pandemic suggest that the cholinergic anti-inflammatory system and the RAS system could converge at some point in the pathways inducing lung inflammation. Understanding such a convergence could help to disentangle the intricate relationship between tobacco smoking and the risk of COVID-19, facilitating new therapeutic approaches for this disease and unveiling more general issues on the regulation of inflammation in lung diseases.

## The Cholinergic Anti-Inflammatory System in Pulmonary Inflammation

Neuronal type nAChR are pentameric receptor-operated cationic channels constituted by the homomeric or heteromeric assembly of nine different isoforms of α, and three different isoforms of β subunits (Yamada and Ichinose, 2018). Among these different subunits the expression of α3, α4, α5, α7, β2, and β4 has been demonstrated in the lung (Lippi and Henry, 2020), where they form nAChR that can be activated either by the Ach directly synthetized by epithelial lung cells (Kuba et al., 2005) or by the Ach released from vagal terminals that innervate also distal airways (Fox et al., 1980). In recent years attention has been focused on homomeric α7 receptors (Hajiasgharzadeh et al., 2019). These rapidly desensitizing, highly Ca2^+^ permeable nAChRs, with the unusual property of responding not only to Ach but also to choline, were first identified in the lung by Wang et al. (2001) who demonstrated their presence in human bronchial epithelial cells. Hollenhorst et al. (2012) demonstrated that nicotine binding to nAchR is effective in activation of transepithelial ion transport in mouse tracheal epithelium involving adenylyl cyclase activity. Kumar et al. (2020) studied many subtypes of nAChRs, such as α3β4, able to increase Ca^2+^ release from intracellular store in the mouse trachea. Studies in α7 nAChR ko mice, showing enhanced tissue inflammation in response to different inflammogens, support the hypothesis that these receptors could negatively modulate the inflammatory response (Su et al., 2010). The cholinergic regulation of macrophage function through nAChR α7 is part of the wider cholinergic anti-inflammatory pathway operated by vagal nerves in the modulation of tissue inflammation (MacKenzie, 2011). In this contest, an anti-inflammatory effect was observed in the lung where it could represent an interesting target for the treatment of both acute and chronic inflammatory diseases (Su et al., 2010; Brégeon et al., 2011; Yamada and Ichinose, 2018). Recent studies indicate that protection from inhaled pathogens evolved in the airways through mucociliary clearance and cough. The protective respiratory reflexes to locally released bacterial bitter “taste” substances are most probably initiated by tracheal brush cells (BC), able to synthetize Ach, effective in stimulating nAChR, and several other intracellular signal molecules (Hollenhorst et al., 2020). In particular, nicotinic stimulation of α3β4-nAChR acutely increases particle transport speed (PTS) on the mucosal surface to the same extent as the established strong activator ATP (Perniss et al., 2020). Krasteva et al. (2011) identified cholinergic chemosensory cells in mouse trachea, able to release Ach and reduce the breathing frequency. These brush cells, as cholinergic sensors of the chemical composition of the lower airway luminal microenvironment, are directly linked to the regulation of respiration and to the immune system modulation. Khani et al. (2020) highlighted the key role played by nicotinic agonists against cytokine storm induced by COVID-19.

## The RAS System in Pulmonary Inflammation

It has been well known that RAS participates not only to the regulation of arterial blood pressure but also in tissue inflammation. By acting on AT1 receptors (Kummer et al., 2008), Angiotensin II (Ang II), indeed, induces free radical generation, activates dendritic cells, stimulates synthesis and release of proinflammatory and chemoattractant cytokines, promotes the expression of endothelial adhesion molecules and leukocyte margination and migration in tissues (Benigni et al., 2010). Similar effects have been demonstrated also in the lung where Ang II promotes free oxygen radical formation (Wang et al., 2017), enhances vascular permeability and edema formation (Zhang and Sun, 2005), and contributes to tissue damage and remodeling by promoting the apoptosis of alveolar epithelial cells (Wang et al., 1999) and the proliferation of fibroblasts (Marshall et al., 2000). Ang II proinflammatory effects are homeostatically counteracted by another RAS peptide, Angiotensin (1-7) [Ang-(1-7)] that is generated by the alternative metabolism of Ang I or Ang II by ACE2 (Silva and Teixeira, 2016), an 805 amino acids transmembrane metallopeptidase, cloned 20 years ago by Tipnis et al. (2000). More specifically, this enzyme removes a single residue from Ang II to generate Ang-(1–7) and a single residue from Ang I to yield Ang-(1–9) which is further processed to generate Ang-(1-7) (Donoghue et al., 2000). Acting on AT2 receptors and Mas receptors, Ang-(1-7) counteracts Ang II activity by inducing anti-proliferative, anti-inflammatory and antifibrotic effects as it has been demonstrated in animal models of several inflammatory disorders including rheumatoid arthritis, diabetic nephropathy, hepatic fibrosis and lung diseases such as asthma, pulmonary fibrosis and the respiratory distress syndrome (Benter et al., 2008; El-Hashim et al., 2012; Galvão et al., 2019; Magalhães et al., 2019). Interestingly, beneficial effects, probably dependent from increased Ang-(1-7) generation have been demonstrated in piglets with lipopolysaccharide-induced lung injury upon treatment with recombinant ACE-2 (Treml et al., 2010): this enzyme has also been tested in a pilot trials in humans with ARDS (Khan et al., 2017).

A new role for ACE2 in pulmonary diseases has been demonstrated by studies on two new human coronaviruses, SARS-CoV and SARS-CoV2. SARS-CoV causes the SARS epidemics that determined about 8400 cases and more than 800 deaths in 2003/2004 (Staats et al., 2020). SARS-CoV2 is responsible for the ongoing COVID-19 pandemic that at the time of writing already caused 8.52 million of cases and more than 450.000 deaths in the World (World Health Organization (WHO), 2020). Both these viruses enter target cells in the airways and diffuse into susceptible organs, such as the brain, upon binding of their surface spike protein (S) to plasma membrane ACE2. More specifically, after binding to ACE 2 the S protein is cleaved by the Transmembrane Serine Protease 2 (TMPRSS2); its carboxy-terminal S2 domain is released, facilitating the fusion of viral membrane with the plasma membrane of the target cell (Hoffmann et al., 2020). ACE2 has a crucial role in the pathophysiology of SARS-CoV and SARS-CoV2 infections, as the key factor for virus penetration; however, it also participates to the pathophysiological mechanisms of these diseases in a way related to its physiological activity in the RAS modulation. It has been suggested, indeed, that ACE2 activity could by impaired after S protein binding, with consequent imbalance of the RAS mechanisms, facilitating the proinflammatory activities of Ang II. Upon exposure to a fusion protein of the SARS-CoV S protein and the immunoglobulin Fc fragment, Su et al. (2010) showed that the surface density of ACE2 is significantly lowered both in HEK-293 overexpressing this enzyme and in Vero E6 cells endogenously expressing ACE2. The mechanism suggested for this S protein effect is receptor-dependent endocytosis and the same has been hypothesized in susceptible cells challenged with SARS-CoV. Hajiasgharzadeh et al. (2019) showed that SARS-CoV also promotes TACE-dependent cleavage of ACE2 in plasma-membrane adding to ACE2 shedding and to a decrease of local ACE2 availability and activity. A demonstration, that S-protein-dependent loss of ACE2 activity could facilitate the progression of lung inflammation, was given by experiments showing that the S-Fc fusion protein causes functional imbalance of the RAS mechanisms worsening the lung damage induced by acid aspiration in mice: this worsening effect is prevented with AT1 receptor antagonists (Su et al., 2010). The same mechanism has been suggested during COVID-19 and could critically contribute to the devastating progression of this disease.

## Does the Cholinergic Anti-Inflammatory Pathway Regulate Pulmonary RAS Hints From the COVID-19 Pandemics?

The hypothesis that cholinergic mechanisms could modulate the RAS in the lung has been proposed to explain the effect of tobacco smoking on the risk of contracting COVID-19 (Changeux et al., 2020). A high prevalence of smokers among the COVID-19 patients with the most serious disease was reported at the beginning of the pandemic in China (Guan et al., 2020). Since then, several studies analyzed the relationship between tobacco smoking and the severity of COVID-19 infection with contradictory results. For instance, Vardavas and Nikitara (2020) showed that tobacco smoking increases the risk of a serious course of the disease; Lippi and Henry (2020) reported a non-significant trend for such association and Farsalinos et al. (2020) observed that in 13 studies from China the prevalence of smokers among patients with COVID was significantly lower than in general population. By examining 15 published studies, Alqahtani et al. (2020) found that the disease was much more serious in smokers than in non-smokers, although the prevalence of smokers and patients with Chronic obstructive pulmonary disease (COPD) was lower among patients with COVID-19. Another meta-analysis of data from 18 studies showed that smokers were statistically less likely to be hospitalized for serious COVID-19 (OR = 0.18, 95% CI: 0.14–0.23, *p* < 0.01) (González-Rubio et al., 2020). These data could suggest that tobacco smoking reduces the probability of contracting COVID-19 but worsens the prognosis of the disease when contracted. Whilst the effect of cigarette smoking on COVID-19 remains uncertain, experimental evidence supports the hypothesis that it increases ACE-2 activity as demonstrated in the peripheral blood of healthy volunteers (Kimura et al., 2019) and in isolated rat lungs (Bakhle et al., 1979). Moreover, it has been shown that the exposure to cigarette smoke increases the activity of both ACE and ACE2 activities in the lung in mice (Hung et al., 2016), and that the expression of ACE2 is higher in the small airways of human patients with COPD and in current smokers when compared with healthy subjects and former smokers (Alqahtani et al., 2020; Leung et al., 2020). Since tobacco smoke has many chemical components besides nicotine, it could affect ACE or ACE2 expression by multiple mechanisms, not necessarily limited to the nicotine-dependent stimulation of nAChR. For instance, it could induce tissue inflammation, which enhances the release of cytokines known to significantly increase ACE2 expression in the lung (Wösten-van Asperen et al., 2008), and selectively increases the number of the epithelial cells which express ACE2 including alveolar type I, globet and club cells (Smith et al., 2020). However, it is likely that nicotine by itself may exert a role in this process considering that it induces ACE2 upregulation in cultured human bronchial epithelial cells and this effect is blunted by the selective nAChR blocker, α-bungarotoxin, and by anti-α7 siRNAs (Russo et al., 2020). Nicotine contained in smoke could be, therefore, acting as an activator of the cholinergic anti-inflammatory system in the lung, which could be beneficial in COVID-19 (Tizabi et al., 2020; Qin et al., 2021). Indeed, although cholinergic stimulation is expected to increase the density of the SARS-CoV2 receptor ACE2, the higher activity of this enzyme could reduce Ang II-induced proinflammatory status mainly by enhancing the levels of its functional antagonist Ang-(1-7) (Magalhaes et al., 2020). A further argument to suggest that nicotinic receptor stimulation could be beneficial in COVID-19 is that it could counteract the inhibitory effect of SARS-CoV2 on nicotinic receptors. As a matter of fact, it has been observed the S protein of SARS CoV2 shows sequence analogies with the nAChR blocker α-bungarotoxin (Kimura et al., 2019), and *in silico* studies showed that it can bind to nAChR and that several nAChR agonists including nicotine can effectively prevent this binding (Alexandris et al., 2020; Lagoumintzis et al., 2021). The inhibitory effects of SARS-CoV2 on nAChR are expected to imbalance the equilibrium between Ang II and Ang-(1-7) in favor of the former, hence promoting lung inflammation, whereas cholinergic stimulation could bring back to normal the Ang II/Ang-(1-7) balance. Under this respect, it is worth noting that beneficial effects of enhancing ACE-2 have been demonstrated in a single patient with severe COVID-19 (a trial is ongoing) who was given human recombinant soluble ACE2 with the double rationale of blocking SARS-CoV2 spike proteins and of lowering Ang II at the same time enhancing Ang-(1-7) (Zoufaly et al., 2020). Because of its well-known toxicity for the airways, cigarette smoke cannot be recommended to enhance cholinergic tone, but both pharmacological and non-pharmacological tools can be used to this aim. For instance, clinical trials are ongoing to evaluate the efficacy in COVID-19 patients of nicotine patches (the NICOVIDREA trial, NCT04598594), pyridostigmine bromide (the PISCO trial) (Fragoso-Saavedra et al., 2020) or vagal nerve stimulation (NCT04379037; Fudim et al., 2020; Staats et al., 2020). The results of these studies are eagerly expected as they could provide the clinical evidence, which is still missing (Wenzl, 2020), that cholinergic stimulation could be helpful in COVID-19 patients.

## Conclusions and Future Perspectives

**FIGURE 1:**
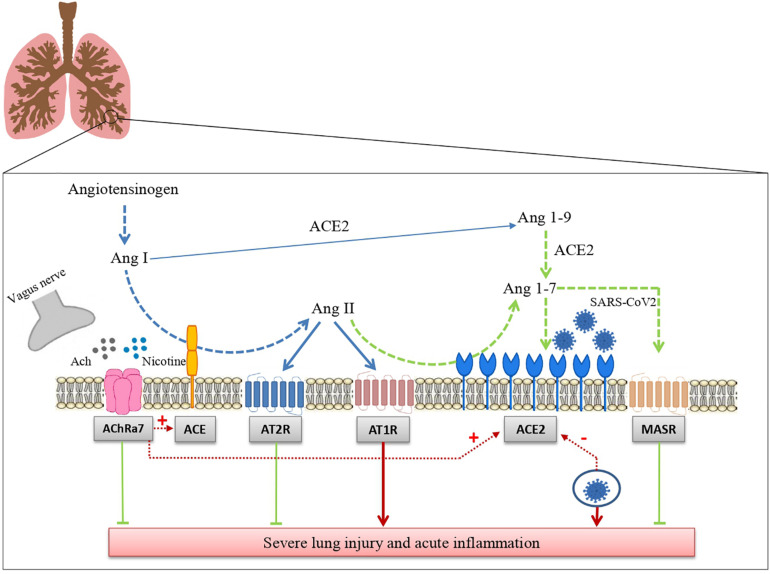
Interaction between the renin angiotensin system and the cholinergic anti-inflammatory pathway in SARS-CoV-2 lung infection. The drawing shows how the renin angiotensin system and the cholinergic anti-inflammatory pathway modulate tissue inflammation in lungs infected with SARS-CoV-2 and how these two systems interact with each other (see the text for further details). Ang I: Angiotensin I; Ang II: angiotensin II; Ang-(1–7): angiotensin 1-7; Ang-(1–9): angiotensin 1-9; ACE: angiotensin-converting enzyme; ACE2: Angiotensin-converting enzyme 2; AT1R: Angiotensin Receptor 1; AT2: Angiotensin Receptor 2; MASR: Mas receptors.

In conclusion, we have summarized the data suggesting that RAS and the cholinergic anti-inflammatory system may represent two major regulatory mechanisms of inflammation in the lung. We have reported that ACE2 acts as a receptor for SARS-CoV2 and the still controversial effect of tobacco smoking leading to a cholinergic hypothesis for COVID-19. This hypothesis postulates that nicotine acting through nAChR modifies risk and prognosis of this disease by changing ACE2 levels (Figure 1). Besides the potential relevance for the pharmacological treatment of COVID-19, that remains to be established, the hypothesis that RAS and the cholinergic system may functionally interact certainly deserves further attention because of its potential implications for basic lung physiology and for the pathophysiology of pulmonary diseases.

## Author Contributions

MDM, MCa, and BG: conceptualization, supervision, and writing. MDM, MCh, and BDC: writing. MS, SD, AC, and BG: review and editing. All authors have read and agreed to the published version of the manuscript.

## Conflict of Interest

The authors declare that the research was conducted in the absence of any commercial or financial relationships that could be construed as a potential conflict of interest.

## Abbreviations

ACE: Angiotensin-converting enzyme
ACE-2: Angiotensin-converting enzyme 2
Ach: Acetylcholine
Ang II: Angiotensin II
Ang-(1-7): Angiotensin-(1–7)
Ang-(1–9): Angiotensin-(1–9)
AT1-R: Angiotensin II receptor type 1
AT2-R: Angiotensin II receptor type II
COPD: chronic obstructive pulmonary disease
COVID-19: coronavirus disease 2019
Ko: knockout
Mas-R: Mas-receptor
nAChR: nicotinic acetylcholine receptors
RAS: Renin-angiotensin system
SARS-CoV2: severe acute respiratory syndrome coronavirus 2.
